# Influenza Infection in Wild Raccoons

**DOI:** 10.3201/eid1412.071371

**Published:** 2008-12

**Authors:** Jeffrey S. Hall, Kevin T. Bentler, Gabrielle Landolt, Stacey A. Elmore, Richard B. Minnis, Tyler A. Campbell, Scott C. Barras, J. Jeffrey Root, John Pilon, Kristy Pabilonia, Cindy Driscoll, Dennis Slate, Heather Sullivan, Robert G. McLean

**Affiliations:** US Department of Agriculture National Wildlife Research Center, Fort Collins, Colorado, USA (J.S. Hall, K.T. Bentler, S.A. Elmore, J.J. Root, J. Pilon, H. Sullivan, R.G. McLean); Colorado State University College of Veterinary Medicine and Biomedical Sciences, Fort Collins (G. Landolt, K. Pabilonia); Mississippi State University, Starkville, Mississippi, USA (R.B. Minnis); US Department of Agriculture National Wildlife Research Center, Kingsville, Texas, USA (T.A. Campbell); US Department of Agriculture Wildlife Services, Moreley, Virginia, USA (S.C. Barras); Maryland Department of Natural Resources, Oxford, Maryland, USA (C. Driscoll); US Department of Agriculture Wildlife Services, Concord, New Hampshire, USA (D. Slate); 1 Current affiliation: US Geological Survey National Wildlife Health Center, Madison, Wisconsin, USA.

**Keywords:** Influenza, wildlife, host, seroprevalence, infection, receptors, risk, reassortment, raccoon, research

## Abstract

Raccoons (*Procyon lotor*) are common, widely distributed animals that frequently come into contact with wild waterfowl, agricultural operations, and humans. Serosurveys showed that raccoons are exposed to avian influenza virus. We found antibodies to a variety of influenza virus subtypes (H10N7, H4N6, H4N2, H3, and H1) with wide geographic variation in seroprevalence. Experimental infection studies showed that raccoons become infected with avian and human influenza A viruses, shed and transmit virus to virus-free animals, and seroconvert. Analyses of cellular receptors showed that raccoons have avian and human type receptors with a similar distribution as found in human respiratory tracts. The potential exists for co-infection of multiple subtypes of influenza virus with genetic reassortment and creation of novel strains of influenza virus. Experimental and field data indicate that raccoons may play an important role in influenza disease ecology and pose risks to agriculture and human health.

## Abstract 2

Raccoons can transmit avian and human influenza Influenza Infection in Wild Raccoons

The primary reservoirs of avian influenza (AI) are wild birds in the orders Anseriformes (ducks, geese, and swans) and Charadriiformes (gulls, terns, and shorebirds). In these hosts, low-pathogenic forms of the virus typically cause little or no apparent disease, however, large quantities of virus are shed in fecal matter. AI virus is relatively stable in water and can remain viable for up to 200 days, depending on temperature and other environmental factors (*1*). Thus, bodies of water and adjacent shorelines that wild birds use can become potentially contaminated, increasing the likelihood of subsequent exposure of avian and non-avian species to AI virus.

The preference of influenza viruses for different cellular receptors and the presence and distribution of those receptors in the host are important factors involved in determining host range and tissue tropism (*2*). Humans are not typically infected by AI virus because receptors for this virus are distributed in tissues that are located predominantly in the lower respiratory tract. As such, these receptors are not as accessible as human type receptors found in the upper respiratory tissues and require more intimate contact for transmission. Swine are considered important intermediate hosts between birds and humans because they are frequently infected by avian and human influenza viruses (*3*). This finding underscores the potential for genetic reassortment that can create new, possibly more virulent subtypes.

Other non-avian hosts of AI virus include mink, harbor seals, pilot whales, dogs, cats, and horses (*4*). These species were found to be competent hosts only after attracting attention because of severe death or illness (*4*). Wild mammals often reside in the same habitats as waterfowl, feed in the same agricultural areas, wallow and swim in the same bodies of water, and prey on and scavenge dead birds for food. Therefore, ample opportunities exist for free-ranging wild mammals to be exposed to AI by contact with waterfowl and their environment. Many of these species are highly mobile and have large home ranges that can include agricultural operations, wetlands, and human residences. Humans are frequently unaware of their presence, and wild mammals have the potential to contract AI from waterfowl or their environment and to then transmit AI to domestic animals or humans. To date, no studies have systematically examined wild mammalian species, particularly peridomestic mammals, for exposure to AI, their ability to become infected, and their reassortment potential. This knowledge is critical for accurate risk assessments of low pathogenic and highly pathogenic AI to agriculture and human health.

Raccoons (*Procyon lotor*) are widespread and common in riparian, wooded, and suburban settings over much of North America (*5*). Previously, antibodies against AI virus (H4N6) were found in 1 raccoon in Pennsylvania (J. Hall, unpub. data). This finding led us to conduct the present study in which we examined wild populations of raccoons from various regions of the United States for antibodies to influenza virus. Experimental infections of raccoons with avian and human influenza viruses were performed to determine viral shedding, transmission, and immune response. The abundance and distribution of avian or human influenza virus cellular receptors in respiratory tissues was analyzed to determine potential for co-infection and possible reassortment of influenza virus strains in this host. These data provided insight into the complexity of influenza disease ecology and the overlooked, potentially important roles of peridomestic wildlife in transmission cycles.

## Materials and Methods

### Field Sample Collection

Blood was opportunistically collected from wild raccoons taken during population control operations in various counties/parishes in Texas, Wyoming, Louisiana, California, and Maryland. Raccoons from northwestern Georgia were sampled as part of the US Department of Agriculture Cooperative National Oral Rabies Vaccination program. Raccoons in Colorado were captured for this, and other studies, in and around Fort Collins, Colorado. Blood samples were obtained by cardiac or jugular puncture, allowed to clot, and centrifuged to separate serum from cellular blood components. Serum was transferred to fresh cryovials and stored frozen (–20°C) until transport to the National Wildlife Research Center in Fort Collins, where they were stored at –80°C until analysis.

### Screening for Antibodies to Influenza Virus

Agar gel immunodiffusion is a serologic assay used to detect antibodies to influenza viruses. The antigen used in the assay was derived from the matrix and nucleoproteins of AI and is used to detect antibodies to all subtypes of AI. The procedure has been described by Beard (*6*) and was performed by using reagents and the protocol provided by the Center for Veterinary Biologics and National Veterinary Services Laboratories (Ames, IA, USA).

### Determination of Antibody Subtypes for Influenza Virus

Hemagglutination inhibition and neuraminidase inhibition are used to determine subtype identity of influenza antibodies in sera. These procedures are described by Beard (*6*) and were performed at the National Veterinary Services Laboratories.

### Experimental Infection of Raccoons

Ten wild raccoons were live-trapped in and around Fort Collins, Colorado. These animals were transported to the National Wildlife Research Center and held for a 2-week quarantine period, where they were observed daily and judged to be in good overall health on the basis of food intake, behavior, and absence of clinical signs of disease. After 2 weeks, the animals were anesthetized and moved into a biocontainment level 2 facility. Blood samples and nasal and rectal swabs were collected, and animals were placed in individual cages (5 per room). Four animals in each room were inoculated intranasally with 10^5^ 50% egg infectious dose (EID_50_) of AI virus A/CK/AL/75 (H4N8) diluted in 100 μL of viral transport medium. The fifth animal in each room was uninoculated and monitored to determine transmission between animals. To prevent potential fomite transmission, these controls were always handled and sampled first when technicians entered the rooms, and all food and water bowls were cleaned and sanitized in hypochlorite solution each day. Each animal was provided food and water ad libitum and observed daily for illness, behavior, and general welfare. To avoid excessive handling and anesthesia, serum samples, nasal and rectal swabs, and rectal temperatures were collected from each group of 5 raccoons on alternating days for 14 days. Blood samples were obtained by jugular puncture. Nasal and rectal swabs were collected by using dacron-tipped applicators placed into viral transport media after swabbing. All samples were stored at –80°C until analyzed.

Subsequently, a second cohort of 6 raccoons was captured in Fort Collins, quarantined, and placed into biocontainment. Four raccoons were intranasally inoculated with 10^5^ EID_50_ of human influenza virus (A/Aichi/2/68 [H3N2]) and were sampled and monitored as described above. Two uninoculated raccoons were housed in cages adjacent to inoculated animals to assess transmission. All animal handling, trapping, and experimental infections were performed following Institutional Animal Care and Use Committee and institutional biosafety protocols, guidelines, and approval.

### Reverse Transcription–PCR

Viral RNA was extracted from nasal swabs by using the QIAamp Viral RNA Mini Kit (QIAGEN, Valencia, CA, USA), following manufacturer’s instructions. Viral RNA from rectal swabs was extracted by using the same procedure with addition of half of an Inhibitex tablet (Stool Extraction Kit; QIAGEN) to remove PCR inhibitors. Real-time reverse transcription–PCR (RT-PCR) was performed following the procedure of Spackman et al. (*7*). Samples were compared with standard curves generated from known concentrations of AI, extracted, and amplified by using the same procedures. Results are expressed as EID_50_ equivalents. These procedures were used to analyze environmental samples (feces and water) as part of the national surveillance for highly pathogenic AI (*8*).

### Detection of Influenza Virus Cellular Receptors in Raccoon Respiratory Tissues

Airway tissue sections were collected from 5 adult raccoons (humanely killed in a different study) from 7 standardized locations (nasal mucosa; larynx; upper, middle, and lower trachea; bronchus; lung), fixed in formalin, and embedded in paraffin. Airway tissues were cut into 5 μm–thick sections, mounted on 3-aminopropyltrethoxy-silane–coated slides, deparaffinized in xylene, and rehydrated in alcohol. For detection of sialic acids (SAs), sections were stained with SAα2,3Gal- and SAα2,6Gal-specific lectins. Briefly, sections were incubated overnight with 250 μL of Western blocking solution (Roche Biochemicals, Indianapolis, IN, USA), washed 3× in Tris-buffered saline, pH 7.6, and incubated with 250 μL of fluorescein isothiocyanate–labeled *Sambucus nigra* lectin (Vector Laboratories, Burlingame, CA, USA) and biotinylated *Maackia amurensis* lectin (Vector Laboratories) overnight at 4°C. After 3 washes in Tris-buffered saline, sections were incubated with Alexa Fluor 594–conjugated streptavidin (Molecular Probes, Inc., Eugene, OR, USA) for 2 hours at room temperature. The sections were washed, counterstained with 4′,6-diamino-2-phenylindole dihydrochloride (Molecular Probes), washed again, and mounted on cover glass. Sections were examined with a fluorescence microscope (Carl Zeiss, Inc., Oberkochen, Germany). Because previous research has demonstrated that equine tracheal epithelial cells predominantly express SAα2,3Gal residues and pig tracheal cells express SAα2,3Gal and SAα2,6Gal residues (*2*), sections of equine and porcine trachea were included as positive controls for each staining procedure.

## Results

### Serologic Survey of Wild Raccoons for Exposure to AI Virus

We screened 730 wild raccoons from California, Texas, Louisiana, Maryland, Wyoming, and Colorado. Of these, 17 (2.4%) had antibodies to AI virus. Table 1 summarizes the raccoon serosurvey and subtyping results from these states. Four (2.4%) of 168 Maryland raccoons in 2004 had antibodies to AI virus with 3 hemagglutinin subtypes represented. Two of these raccoons had antibodies to 2 subtypes, which indicated multiple exposures to AI virus. Colorado and Wyoming also had seropositive raccoons with prevalences of 12.8% and 25%, respectively. Multiple subtypes were present in both populations, and multiple exposures in individual raccoons were observed. However, none of the raccoons from Georgia, Texas, or California showed serologic evidence of exposure to AI virus. These results indicated that wild raccoons are exposed to a variety of AI virus subtypes and seroconvert on the basis of these exposures.

**Table 1:** Exposure to avian influenza virus in wild raccoons in 7 states, United States*

State	Year	No. positive/no. tested (%)	Influenza antibody subtypes (no.)
MD	2004	4/168 (2.4)	H4 + H10† (1), H1 + H10† (1), H4† (2)
	2005	0/13	–
GA	2004	0/366	–
CA	2006	0/46	–
TX	2004	0/40	–
	2006	0/16	–
LA	2004	0/10	–
WY	2004	8/32 (25)	H4N6 (7), H4N2 (1)
CO	2006	5/39 (12.8)	H4N2 + N6 (3), H3† (1), H10N7 (1)

*H, hemagglutinin; N, neuraminidase. †N subtype was not determined because of insufficient sample volume.

### Experimental Infection of Raccoons with AI Virus

To determine whether raccoons are competent hosts for AI virus infection and are capable of shedding and transmitting virus, raccoons were infected with a specific subtype of AI virus (H4N8) and monitored for symptoms of infection and disease. Two of 10 wild-caught raccoons had antibodies to AI virus (Table 1). These animals were included in the infection study because the AI virus inoculum used was a different subtype, but with potential cross-neutralization as a caveat.

Eight raccoons were inoculated intranasally with 10^5.0^ EID_50_ of AI virus (H4N8) and monitored for 14 days postinoculation (dpi). Four (50%) of these animals became infected, as shown by nasal shedding of viral RNA detected by RT-PCR. Two of these animals (256 and 275) shed detectable amounts of virus at only 1 time point (1 dpi). Another raccoon (259) shed virus at least up to 6 dpi, and the other infected raccoon (263) shed for the entire 14 days of the study (Table 2). RT-PCR analyses of rectal swabs showed no detectable viral RNA shed by digestive tracts of infected raccoons (data not shown), which is consistent with influenza being primarily a respiratory disease in mammals (*2*).

**Table 2:** Nasal shedding of avian influenza virus by experimentally inoculated raccoons*

Raccoon ID	Day postinoculation														
	0	1	2	3	4	5	6	7	8	9	10	11	12	13	14
264	–	–		–		–		–		–		–		–	
275	–	0.2		–		–		–		–		–		–	
256	–	1.1		–		–		–		–		–		–	
253	–	–		–		–		–-		–		–		–	
260†	–	–		–		–		–		–		–		–	
262†	–		0.2		0.8		0.9		0.4		–		–		–
258	–		–		–		–		–-		–		–		–
257	–		–		–		–		–		–		–		–
259	–		1.1		0.1		0.4		‡						
263	–		–		0.6		0.2		0.9		0.02		0.4		0.1

*Shedding was determined by real-time reverse transcription–PCR of nasal swabs compared with standard curves generated from avian influenza virus stocks of known concentrations and expressed as log_10_ 50% egg infectious dose equivalents. –, no viral RNA detected. Raccoons 262 and 263 were seropositive for avian influenza virus before inoculation. †Uninoculated raccoons housed in cages adjacent to infected raccoons. ‡Raccoon humanely killed 8 days postinoculation.

One of the 2 uninoculated raccoons housed in cages adjacent (within ≈0.5 m) to inoculated raccoons developed nasal shedding of virus. Every precaution was taken to prevent inadvertent transmission by handling; thus, this animal (262) probably contracted the virus by aerosol from >1 of its infected cohorts. This result indicated that raccoons are capable of transmitting influenza virus from one to another. Given the small amounts of AI virus shed by these raccoons and the timing of infection of this animal, we cannot rule out possible aerosolization of inoculum by adjacent raccoons and transmission by that route.

Three of the 5 raccoons that shed virus developed antibodies to the AI virus (H4N8) isolate, including raccoon 262, which was not inoculated but contracted the virus from adjacent, infected raccoons (Table 3). Raccoon 259 was humanely killed on 8 dpi because of an unrelated physical condition (tooth abscess), presumably before detectable antibodies were produced. Raccoon 256 shed virus only on 1 dpi yet developed detectable antibodies to AI virus (H4N8) by 9 dpi. However, the other raccoon that shed virus on 1 dpi (275) did not develop a detectable immune response, which indicated that virus detected in the swab was probably residual inoculum. Raccoons 263 and 262 had preexisting antibodies to a different subtype of AI virus that did not prevent infection and seroconversion to the other AI virus (H4N8) inoculum.

**Table 3:** Antibody production in raccoons experimentally infected with avian influenza virus (H4N8)*

Raccoon ID	Day postinoculation															Final subtype†
	0	1	2	3	4	5	6	7	8	9	10	11	12	13	14	
264	N	N		N		N		N		N		N		N		
275	N	N		N		N		N		N		N		N		
256	N	N		N		N		N		WP		SP		SP		H4N8
253	N	N		N		N		N		N		N		N		
260‡	N	NS		N		N		N		N		N		N		
262‡	P		P		P		P		P		P		WP		WP	H4N8§
258	N		N		N		N		N		N		N		N	
257	N		N		N		N		N		N		N		N	
259	N		N		N		N		N		¶					–
263	SP		SP		SP		SP		SP		SP		SP		SP	H4N8§

*Antibodies were detected by agar gel immunodiffusion, and final subtyping was determined at the National Veterinary Services Laboratories (Ames, IA, USA). N, negative; WP, weakly positive; SP, strongly positive; NS, not sampled; P, positive. †H, hemagglutinin; N, neuraminidase. ‡Uninoculated raccoons housed in adjacent cages to infected raccoons. §These raccoons had preexisting antibodies (262:H10N7, 1:10; 263:H4N6 1:40). ¶Raccoon humanely killed 8 days postinoculation.

We observed no overt clinical signs of disease in these animals. Rectal temperatures showed no obvious trends and were probably confounded by stresses of anesthesia and handling. Most of the animals appeared lethargic, possibly because of confinement and manipulations occurring during daytime (raccoons are nocturnal). All other animals ate and drank well and most gained weight over the course of the experiment (data not shown).

### Influenza Virus Receptors in Raccoons

The predominant receptor for AI virus is SA linked α2,3 to galactose. In waterfowl, these receptors are located primarily in intestinal epithelium, which is why AI is primarily a disease of the digestive tract in avian species. In contrast, humans have SA linked α2,6 to galactose that is located predominantly in the respiratory system (*2*). Tissues from raccoon respiratory tracts were examined for avian and human influenza virus receptors by staining with lectins specific for each type of receptor (Figure). Raccoons have both receptor types in their respiratory systems, similar to swine but with uneven distribution among tissues. In the upper trachea epithelium, the overwhelmingly predominant receptor is the human type SAα2,6 (Figure, panel A). As one examines tissues from deeper in the respiratory tract, increasing amounts of the avian SA α2,3 receptor are found until the 2 types of receptors are in roughly equal amounts in the lungs (Figure, panels B, C, and D).

**Figure:**
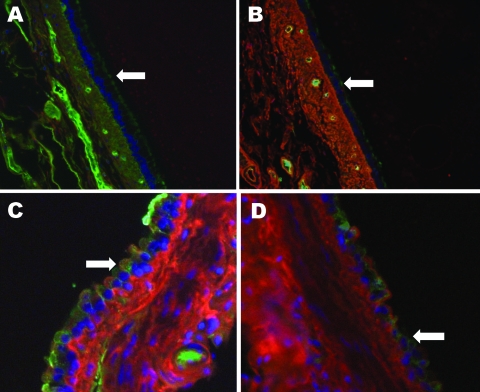
Raccoon respiratory tissues stained with lectins specific for sialic acids (SAs) with α2,6- and α2,3-linkages. A) Upper trachea; B) lower trachea; C) bronchus; D) bronchiole. Arrows indicate endothelial lining of the tissues indicated. Green staining shows a reaction with fluorescein isothiocyanate–labeled *Sambucus nigra* lectin, which indicates SAs linked to galactose by an α2,6-linkage (SAα2,6Gal). Red staining shows a reaction with biotinylated *Maackia amurensis* lectin (detected with Alexa Fluor 594–conjugated streptavidin), which indicates an SAα2,3Gal linkage. Tissues were counterstained with 4,6,-diamidino-2-phenylindole dihydrochloride. Original magnification ×40 in panels A, B, and D and ×100 in panel C.

### Experimental Infection of Raccoons with Human Influenza Virus

The presence and distribution of human type receptors in raccoons led us to infect a new cohort of raccoons with human influenza virus (H3N2). Daily monitoring showed that inoculated animals shed virus nasally for up to 8 dpi (Table 4, Figure). The amounts of virus shed were larger than in the AI experimental infection study but no transmission to either co-housed, virus-free raccoon was detected. All 4 inoculated animals subsequently developed antibodies against this virus by 14 dpi (data not shown). One raccoon (272) shed small amounts of virus rectally (0.25 EID_50_ equivalents) on 5 dpi, but no other rectal shedding of virus was detected. As with AI virus infection, no obvious clinical signs of disease were observed in these animals. Infected raccoons were also capable of shedding moderate amounts of human influenza virus, although no transmission to virus-free animals was observed.

**Table 4:** Nasal shedding of human influenza virus by experimentally inoculated raccoons*

Raccoon ID	Day postinoculation														
	0	1	2	3	4	5	6	7	8	9	10	11	12	13	14
265	–		0.2		–		–		–		–		–		–
267	–		3.2		2.4		0.3		0.3		–		–		–
269†	–		–		–		–		–		–		–		–
268†	–	–		–		–		–		–		–		–	–
271	–	–		2.0		0.5		0.2		–		–		–	–
272	–	0.2		0.2		2.0		1.2		–		–		–	–

*Shedding was determined by real-time reverse transcription–PCR of nasal swabs compared with standard curves generated from avian influenza virus stocks of known concentrations and expressed as 50% log_10_ egg infectious dose equivalents. –, no viral RNA detected. †Uninoculated raccoons housed in cages adjacent to infected raccoons.

## Discussion

The ecology of AI is complicated. Knowledge of the roles of wild birds and mammals in the epidemiology of the disease and how viral reassortants and variants arise are critical for the planning and preparation of future pandemics, vaccine development, and meaningful human health and agricultural risk assessments (*9*,*10*). However, other than a survey of small rodents in Pennsylvania, New Jersey, Maryland, and Virginia after an outbreak of influenza caused by virus subtype H5N2 in 1983–84 (*11*), no systematic investigation of wild mammals in influenza disease ecology has been performed.

Raccoons can carry a variety of etiologic agents. In Florida, raccoons are known to harbor 132 parasites, disease agents, and environmental contaminants, more than any other species of wild mammal (*12*). Viral diseases include rabies, canine distemper, pseudorabies, and poxvirus disease. To this list we can add West Nile virus (*13*,*14*) and now, from this study, avian and human influenza viruses.

The serologic survey of raccoons for AI virus exposure showed geographic variation in prevalence. AI in wild birds is relatively common; as much as 30% of the local waterfowl population can be infected (*15*). Raccoons often reside in these areas and can contact AI virus from their food and environment. However, the premise that areas of high waterfowl concentrations promote high antibody prevalence in raccoon populations was not always supported by these data. Raccoons in Georgia were sampled from the northwestern corner of the state, where wild fowl populations are small, and the prevalence of antibodies was 0%. In Maryland, which has one of the highest populations of overwintering and migrating waterfowl on its east coast (*16*), the prevalence of antibodies was 2.4%. Thus, data from these 2 states were logical on the basis of the waterfowl population size. However, Texas and California, with large seasonal populations of waterfowl, showed no evidence of AI virus exposure in raccoons. Wyoming and Colorado, with relatively small waterfowl populations, had the highest exposure rates of any states examined (25% and 12.8%, respectively). The reasons for higher prevalences in Wyoming and Colorado are unclear but may be related to concentrations of raccoons and waterfowl in riparian corridors in these semi-arid areas.

Wild waterfowl are the primary natural reservoir of AI virus, and different subtypes to which these raccoons were exposed are relatively common in avian populations (*17*–*22*). Clearly, raccoons are exposed to AI virus in the wild, and experimental studies confirm they can become infected with this virus and shed virus capable of infecting healthy animals. Also, we showed that raccoons can become infected with human influenza virus and shed moderate amounts of virus. The higher amounts of human influenza virus shed by raccoons than AI virus may indicate that human influenza virus is better adapted to mammalian physiology. The fact that we detected measurable levels of viral shedding with avian and human influenza viruses in infected raccoons is important. If one considers that only 2 uninfected raccoons were available to detect transmission of human influenza virus in this study, the fact that we did not detect transmission does not rule out the possibility that human influenza virus is also capable of being transmitted by raccoons and warrants additional research.

The abundance and distribution of avian and human influenza receptors found in raccoon tissues are similar to those in human respiratory tracts (*23*,*24*). The presence of human and AI virus receptors in raccoon respiratory systems creates the possibility of co-infection with multiple types of influenza virus and, as in swine, genetic reassortment and creation of new, possibly highly virulent strains are distinct risks.

Risks associated with wild raccoons and influenza are compounded by several factors. Raccoons are highly mobile with relatively large home ranges that include a variety of ecologic landscapes (*5*). They routinely travel between wetlands, forests, agricultural operations, and urban and suburban settings. Consequently, a raccoon that acquired AI virus in a marsh from scavenging a diseased bird could easily transport and transmit the virus to poultry and swine operations and to residential areas.

Raccoons apparently are not adversely affected by low pathogenic AI or human influenza viruses and thus remain active and potentially able to transmit virus over large areas. Because of their nocturnal habits, raccoons can be largely invisible to humans but can achieve large population densities. In fact, in some areas more raccoons can inhabit suburban areas than rural areas, reaching >90 raccoons/km^2^ (*25*,*26*).

In summary, the raccoon, a common, peridomestic, wild mammal is capable of becoming infected, transporting, and potentially transmitting avian and human influenza viruses. The risks associated with raccoons and influenza to agriculture and human health are unknown but clearly warrant further research. These results underscore the importance of investigating the roles of other peridomestic species in the disease ecology of influenza.
